# Degradation by Electron Beam Irradiation of Some Elastomeric Composites Sulphur Vulcanized

**DOI:** 10.3390/ma16062152

**Published:** 2023-03-07

**Authors:** Elena Manaila, Gabriela Craciun, Ion Bogdan Lungu, Marius Daniel Dumitru Grivei, Stelescu Maria Daniela

**Affiliations:** 1 Electron Accelerators Laboratory, National Institute for Laser, Plasma and Radiation Physics, 409 Atomistilor St., 077125 Magurele, Romania; 2 Multipurpose Irradiation Facility Center—IRASM, Horia Hulubei National Institute for R&D in Physics and Nuclear Engineering, 30 Reactorului St., 077125 Magurele, Romania; 3 National R&D Institute for Textile and Leather—Leather and Footwear Research Institute, 93 Ion Minulescu St., 031215 Bucharest, Romania

**Keywords:** composites, natural rubber, plasticized starch, vulcanization accelerators, electron beam, irradiation, degradation

## Abstract

Composites based on natural rubber and plasticized starch obtained by the conventional method of sulfur cross-linking using four types of vulcanization accelerators (Diphenyl guanidine, 2-Mercaptobenzothiazole, N-Cyclohexyl-2-benzothiazole sulfenamide, and Tetramethylthiuram disulfide) were irradiated with an electron beam in the dose range of 150 and 450 kGy for the purpose of degradation. The vulcanization accelerators were used in different percentages and combinations, resulting in four mixtures with different potential during the cross-linking process (synergistic, activator, or additive). The resulting composites were investigated before and after irradiation in order to establish a connection between the type of accelerator mixture, irradiation dose, and composite properties (gel fraction, cross-linking degree, water absorption, mass loss in water and toluene, mechanical properties, and structural and morphological properties). The results showed that the mixtures became sensitive at the irradiation dose of 300 kGy and at the irradiation dose of 450 kGy, and the consequences of the degradation processes were discussed.

## 1. Introduction

If, by accident or not, elastomeric waste ends up in the environment, their degradation is a very slow and polluting process. Additionally, the degradation of elastomers is a complex process that depends mainly on the composition, rubber type, and applied method used. Once taken out of use and thrown away, rubber begins to degrade due to the decomposition of the sulfur bonds in it. That may happen after approximately one year, depending on the type and composition of the rubber waste, as well as on its degree of damage. For example, latex gloves decompose in just few months. However, if they are based on synthetic latex, degradation can occur after many years, or even decades. Synthetic rubber soles, and especially those fixed to the bottom of heavy boots, can take a very long time to degrade, lasting between 50 and 80 years. Used tires degrade the longest in the natural environment, with their degradation period lasting up to 2000 years. Moreover, they contain mixed heavy metals, oils, and other substances with a high pollution potential for soil and air [1,2]. The massive use of vulcanized rubber in many fields of activity results in huge amounts of waste and requires the development of new, faster, cheaper, and more efficient degradation methods. The most known methods of natural rubber degradation/depolymerization are chemical degradation, including metathesis reactions and scission of the double bond, ozonolysis, biodegradation, photodegradation (in sunlight and by UV irradiation), and ultrasonic irradiation [3,4]. Chemical degradation takes place by using hydrogen peroxide in the presence of formic acid [3] and periodic acid [5,6]. A metathesis reaction is catalytically induced in the presence of transition metals, such as tungsten, molybdenum, or rhenium [3], and the double bond cleavage reactions are induced by chemical systems, such as chloranil/iron (III) chloride [3,7] or phenylhydrazine/iron (II) chloride [3,8,9]; however, all of these chemical substances are themselves pollutants for the environment. Ozone degrades vulcanized rubbers through its reactions with the double bonds in the main chain, causing chain splitting and reducing surface resistance [10]. In order to accelerate the degradation process, the reaction with ozone is carried out in the presence of a solvent and at a high temperature [11], thus inducing additional process costs. Biodegradation is carried out in the presence of specific microorganisms, and photodegradation is carried out by means of solar UV radiation and UV irradiation [12]; both methods are non-polluting but long-lasting. The use of microwaves is also described as a devulcanization treatment [13,14]. Therefore, all degradation methods require an input of external energy of a chemical, thermal, photochemical, or biological nature. Thus, ionizing radiation, such as accelerated electron beams, can be used as a source of energy to degrade vulcanized elastomeric materials. In the irradiation of any material with high-energy radiation, excited molecules are formed that become reactive species, such as free radicals, ions, and molecules, which modify the molecular structure of the irradiated material.

Irradiation of a material can generate polymerization reactions, branching of the chain, and cross-linking, as well as degradation and splitting of the molecular chain. Depending on the radiation dose (D, expressed in Gy; a dose of 1 Gy is equivalent to 1 joule of energy deposited in a kilogram of substance), the molecular weight of the material exposed to radiation may increase due to polymerization reactions and/or cross-linking or may decrease due to degradation reactions and molecular chain cleavage [15]. In fact, all these types of reactions (polymerization, cross-linking, and cleavage) take place at the same time, and some may predominate over the others, depending on the composition of the irradiated material and the irradiation dose. The free radicals generated on the polymer chains can participate in other reactions, such as inter/intramolecular combinations, inter/intramolecular disproportionation, and cleavage [16]. If irradiation takes place in the presence of oxygen, the radicals formed favor oxidation reactions and thus, through oxidation, the degradation of the irradiated material occurs. All these reactions lead to structural and functional changes, which in turn lead to an alteration of the physical and mechanical properties of the material, as a result of degradation by irradiation [16,17].

The conventional cross-linking of natural rubber, which involves the use of sulfur and organic vulcanization accelerators, is the best known and most used process because it leads to the obtainment of vulcanizates with very good physical and mechanical properties compared to other vulcanization systems [18]. The characteristics of the sulfur vulcanization process, such as the optimal vulcanization time, the scorching time, and the minimum and maximum torsion moments, are significantly influenced by the type of accelerator used [18]. By their nature, they can determine the nature of the bridges that form between macromolecules, being the main way by which the physical properties of vulcanizates can be modeled. For example, 2-Mercaptobenzothiazole (MBT) and N-Cyclohexyl-2-benzothiazole sulfenamide (CBS) allow the obtainment of vulcanized materials with a good aging behavior; MBT, CBS, and Diphenyl guanidine (DPG) ensure satisfactory tensile strength; and Tetramethylthiuram disulfide (TMTD) ensures stability in heat and improved fragility in cold [18]. In conventional cross-linked rubber mixtures, pairs of accelerators with different actions are generally used as follows: accelerators with synergistic action (the joint effect does not exceed the effect of the most active accelerator but is superior to the additive effect); activators (the joint effect does not exceed the effect of the most active accelerator but is superior to the additive effect); or additives (the effect is obtained at the level of the sum of the effects of the components) [18].

The present paper presents the results obtained in the irradiation-degradation experiments of elastomeric composites based on natural rubber (NR) and plasticized starch (PS) cross-linked with sulfur in the presence of four combinations of vulcanization accelerators: MBT and TMTD resulting mixture A; MBT and DPG resulting mixture B; MBT, DPG and TMTD resulting mixture C; and MBT and CBS resulting mixture D. The irradiation was carried out with an electron beam (EB) of 5.5 MeV at the irradiation doses of 150 kGy, 300 kGy, and 450 kGy. The elastomeric composites were characterized before and after irradiation with EB, and the investigations consisted of determining the mechanical properties, gel fraction, cross-linking degree, water absorption, and mass loss in water and toluene. The structural and morphological changes were highlighted by using a Fourier-transform infrared spectroscopy (FTIR) and a scanning electron microscopy (SEM).

## 2. Materials and Methods

### 2.1. Materials and Sample Preparation

The chemicals used in our study were as follows: *natural rubber* of Crepe 1X type from Sangtvon Rubber Ltd., Nakhon Si Thammarat, Thailand, in the form of white rubber sheets (Mooney viscosity of 67.64 ML (1′+ 4′) at 100 °C, volatile matter content of 0.5%, nitrogen content 0.45%, ash content of 0.25%, and impurity content of 0.026%); *maleic anhydride* for synthesis from Merck KGaA, Darmstadt, Germany and Redox Group Company, Bucharest, Romania, (density of 1.48 g/cm^3^ at 20 °C, and melting point/range: 53–58 °C); *soluble potato starch* purchased from Lach-Ner, Neratovice, Czech Republic (water insoluble substances at 0.28%, loss on drying of 16.9%, and easily biodegradable: BOD_5_—0.6 g/g—and COD—1.2 mg/g); *glycerine* for plasticization purchased from SC Chimreactiv SRL, Bucharest, Romania (density: 1.26 g/cm^3^, purity: 99.5%, and free acidity: 0.02%); *Richon IPPD (4010 NA) N-isopropyl-N-phenyl-phenylene diamine*, an antioxidant, purchased from Dalian Richon Chem Co. Ltd., Dalian, China (molecular mass: 493.6374 g/mol and purity: 98%); *zinc oxide*, a curing activator, purchased from S.C. WERCO METAL S.R.L. Zlatna, Romania (97.1% active ingredient); *stearic acid*, a curing activator, purchased from Merck KGaA Gernsheim, Germany, and Redox Group Company, Bucharest, Romania (density: 0.9408 g/cm^3^ at 20 °C and melting point: 70 °C); *polyethylene glycol PEG-4000*, a coupling agent, purchased from Advance Petrochemicals LTD, Gujarat, India (density: 1.128 g/cm^3^ and melting point: 4–8 °C); *perkadox 40 dibenzoyl peroxide* purchased from AkzoNobel Chemicals, Deventer, The Netherlands (density: 1.60 g/cm^3^, active oxygen content: 3.8%, peroxide content: 40%, and pH of 7); *sulfur*, a cross-linking agent, purchased from Merck KGaA Gernsheim, Germany, and Redox Group Company, Bucharest, Romania (purity: 99% and ash: 3%), and 4 types of vulcanization accelerators [19], as shown in Table 1.

Five types of composites containing natural rubber and plasticized starch were obtained based on the recipes presented in Table 2. The polymeric composites were obtained as in our previous work [20] and cross-linked using sulfur in the presence of the four combinations of vulcanization accelerators.

The compatibilizer maleated NR (NR-g-MA) was obtained by roll mixing 100 phr of natural rubber (NR) with 5 phr of maleic anhydride for synthesis and 0.75 phr of perkadox 40. The resulting mixture was kept at a temperature of 160 °C for 30 min and then used as such [20]. Plasticized starch was obtained based on starch, water, and glycerine in the proportions of 50%, 20%, and 30%, respectively. The mixture was stirred at 50–100 rpm for 15 min at 70 °C. After that, it was kept at room temperature for 1 h, then in the oven at 80 °C for 22 h, and finally at 110 °C for 2 h. Drying was carried out at room temperature for 16 h [21].

The four mixtures, which recipes are presented in Table 2, were obtained on a laboratory roll using the mixing technique. The four stages of preparation were as follows: (stage 1) NR and maleated NR were tied on the roll and homogenized for 3 min; (stage 2) ZnO, stearic acid, PEG 400, and Antioxidant PEG 4010 were added to the obtained mixture, and mixing continued for another 2 min; (stage 3) plasticized starch was added, with mixing for another 10 min; and (stage 4) vulcanization agents (sulfur and curing accelerators) were added, with mixing for 5 min before removing from the roll in the form of square plates. The working parameters on the roll were as follows: friction of 1:1.1 and roll temperature of 30–50 °C. The obtained plates were vulcanized using a hydraulic press at a temperature of 165 °C and a pressure of 150 Mpa. The vulcanization time was determined with the Monsanto rheometer [20].

### 2.2. Sample Irradiation

The four types of composite materials vulcanized with sulfur and vulcanization accelerators were irradiated for the purpose of degradation under atmospheric conditions and at a room temperature of 25 ± 2 °C using an ALID-7 electron accelerator of 5.5 MeV from the National Institute for Laser, Plasma and Radiation Physics, Magurele, Romania; the irradiation doses were set to 150, 300, and 450 kGy. During irradiation, the samples were not covered with a polyethylene film, with the presence of oxygen helping the degradation reactions. The irradiation facility and method have been described in our previous work [22].

### 2.3. Laboratory Tests

#### 2.3.1. Gel Fraction and Cross-Link Density

The gel fraction (G) and the cross-link density (ν) were determined based on the equilibrium swelling measurement in the toluene of the composites and calculated by applying the modified Flory–Rehner equation for tetrafunctional networks. The working method was as follows: Circular samples of 15 mm diameter and 2 mm thickness were initially weighed (*m_i_*) and immersed in toluene until the equilibrium absorption was reached. After 6 days, the swollen samples were removed from toluene, wiped with filter paper to remove excess solvent from their surface, and reweighed (*m_g_*). Weighing was carried out in weighing vials with a ground stopper to avoid evaporation of the solvent during this operation. Then, the samples were left to dry at room temperature for 6 days and in a laboratory oven for 24 h at the temperature of 70 °C. Finally, after cooling, the samples were weighed again (*m_s_*). The gel fraction and the cross-link density were determined using the following equations [23,24]:

(1) $$Gel\;fraction\;=\frac{m_{s}}{m_{i}}\times 100$$

(2) $$\nu =\frac{Ln(1-\nu_{2m})+\nu_{2m}+\chi_{12}\nu_{2m}^{2}}{V_{1}\left(\nu_{2m}^{1/3}-\frac{\nu_{2m}}{2}\right)}$$

where *V*_1_ is the molar volume of the solvent (106.5 cm^3^/mol for toluene); *ν*_2*m*_ is the volume fraction of polymer in the sample at equilibrium swelling; and *χ*_12_ is the Flory–Huggins polymer–solvent interaction term [23,24].

The value of the Flory–Huggins polymer–solvent interaction term ($\chi_{12}$) for the natural rubber–toluene system was 0.393 [24,25]. The volume fractions of polymer in the samples at equilibrium swelling (*ν*_2*m*_) were determined from the swelling ratio *G* as follows:

(3) $$\nu_{2m}=\frac{1}{1+G}$$

(4) $$G=\frac{m_{g}-m_{s}}{m_{s}}\times \frac{\rho_{e}}{\rho_{s}}$$

where *ρ_e_* and *ρ_s_* are the densities of the composite samples and the solvent (0.866 g/cm^3^ for toluene), respectively.

The gel fraction and cross-link density determinations were performed at least 3 times, and the presented results are the averages of the single values obtained.

#### 2.3.2. Mechanical Characteristics

Hardness was measured using a hardness tester according to the ISO 7619-1/2011 on 6 mm thick samples. Tensile strength (σ) and specific elongation (ε) were determined using the Material Testing Machine ProLine Z005 from Zwick-Roell, Ulm, Germany, according to the DIN EN ISO 527-1. The dumb-bell test pieces were of type 4, having an overall length of 35 mm, length of the narrow portion of 12 ± 0.5 mm, test length of 10 ± 0.5 mm, thickness of the narrow portion of 2 ± 0.1 mm, and width of 2 mm. The test speed was 200 mm/min. Determinations were performed at least 5 times, and the presented results are the averages of the single values obtained.

#### 2.3.3. Water and Toluene Uptake and Weight Loss

The samples’ uptake in water and toluene before and after irradiation was evaluated according to the ISO 20344/2011, as in previous work [22], by immersion in the solvents at room temperature (23 ± 2 °C) until the samples no longer absorbed the solvents (uptake at equilibrium). Uptake and weight loss in toluene were evaluated based on the results obtained in the gel fraction and cross-linking degree analysis. The uptake at equilibrium in toluene was reached after 6 days and in water after 100 days, respectively. After the equilibrium was reached, the samples were dried in air for 6 days and then in a laboratory oven at 80 °C for 72 h.

#### 2.3.4. Structural Investigations by Fourier-Transform Infrared Spectroscopy

The structural investigations before and after irradiation were performed by an ATR-FTIR analysis using the Spectrum 100 instrument (Perkin Elmer, Waltham, MA, USA). The FTIR spectra were acquired in the ATR mode in the 4000–650 cm^−1^ range, with 50 scans/sample and a resolution of 4 cm^−1^.

#### 2.3.5. Morphological Investigations by Scanning Electron Microscopy

The samples’ surfaces before and after irradiation were examined by using a FEI/Phillips scanning electron microscope (Hillsboro, OR, USA). The samples, which had been fractured in liquid nitrogen and sputtered with gold palladium, were placed in aluminium mounts and scanned at an accelerating voltage of 30 kV.

## 3. Results and Discussion

All four mixtures subjected to EB irradiation for the purpose of degradation contained MBT, a thiazole, as the main vulcanization accelerator. Thiazoles are medium-fast primary accelerators that present only moderate processing safety. They are the most widely used accelerators in the rubber industry, are activated by the combination of zinc oxide and stearic acid, act as retarders of cure in combination with thiourams of the TMTD type, as in mixture A (Table 2), and combine with guanidines of the DPG type, as in mixture B (Table 2), leading to an increase in the vulcanization speed [19]. DPG is a vulcanization accelerator for natural (0.1–0.5 phr) and synthetic (SBR, NBR, in the range of 0.15–0.75 phr and 0.1–0.5 phr, respectively) rubbers, which activates thiazoles (MBT and MBTS), thiurams (TMTM, TMTD), or sulfenamide (CBTS) and provides a good scorch and storage stability [26]. Mixture C (Table 2) is a combination of the vulcanization accelerators used in mixtures A (MBT + TMTD) and B (DPG + MBT), namely a thiazole, a guanidine, and a thiuram. Thiurams (TMTD) are ultra-fast vulcanization accelerators widely used as secondary accelerators. In mixture C was made a combination between TMTD with a thiazole (MBT) with the purpose of fast curing speed and a high recycling density and a guanidine (DPG) that offers good safety during processing [19]. In mixture D, the vulcanization accelerator MBT is used together with CBS, which is a sulfenamide. The sulfenamide class that includes CBS, TBBS, MBS, DCBS, and others is the most popular in the tire industry due to the delayed action and faster cure rate during the vulcanization of rubber compounds containing carbon black. These accelerators provide a wide range of cross-link densities, depending on the type and dosage of accelerator used, and exhibit flat and reversion-resistant cure [19].

### 3.1. Gel Fraction and Cross-Link Density

Before the EB irradiation, the control samples vulcanized with sulfur in the presence of the accelerators were analyzed in order to determine the gel fraction. All the combinations of vulcanization agents used resulted in gel fractions higher than 98%. The increase of gel fractions depending on the type of vulcanization agent used (other than MBT, that was common to all four mixture) was: CBS in mixture D > (DPG + TMTD) in mixture C > TMTD in mixture A > DPG in mixture B.

**Figure 1:**
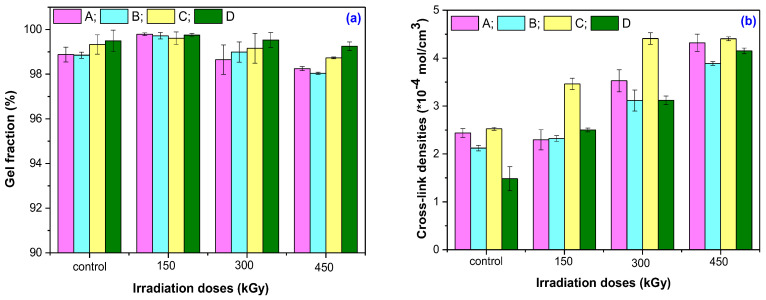
Gel fraction (**a**) and cross-link density (**b**) variation as a function of EB irradiation dose and mixture type.

Figure 1a,b and Table 3 show the results obtained after the investigation of the gel fraction and cross-link density in the irradiated samples. The results presented in Table 3 are reported to be corresponding to the non-irradiated sample.

As can be seen from Figure 1a and Table 3, the irradiation dose of 150 kGy is the one at which the gel fraction shows the highest values. It looks like at 150 kGy, the vulcanization process still continues. By increasing the irradiation dose to 300 kGy and 450 kGy, the gel fraction starts to decrease. The cross-linking bonds already formed between the molecular chains of the natural rubber or between the natural rubber and the filler (starch) begin to break. Thus, low-molecular-weight products are formed, which are the products that will be eliminated as a result of the immersion in the solvent (toluene). The results obtained after the investigation of changes in the cross-link densities with increasing irradiation dose support the above findings. As can be seen from Figure 1a,b, the decrease in the gel fraction is accompanied by an increase in the cross-link densities for all mixtures. The decrease in the gel fraction, as a function of the irradiation dose and the mixture type at an irradiation dose of 450 kGy, is as follows: D < C < A < B. For the cross-link densities, at the same irradiation dose of 450 kGy, the order is as follows: C < A < B < D. Mixture D shows the highest increase in cross-link density and the lowest loss in gel fraction with an increase in the irradiation dose. An analysis of the results presented in Figure 1a,b and Table 3 allows us to state that mixtures C (MBT + DPG + TMTD) and D (MBT + CBS) are the most resistant to EB irradiation from the point of view of gel fraction and cross-link density. The cross-link density increases with increasing irradiation dose for all four mixtures. At the same time, the gel fraction decreases, which means that the absorption percentage of the solvent also decreases. The increase in cross-link density associated with the decrease in solvent absorption still shows a compact structure of the composite. The limitation of molecular movement reduces the ability of the NR chain to expand upon solvent diffusion in the matrix, which explains the decrease in solvent absorption [27,28]. In addition, the formation of new cross-links as a result of the degradation/devulcanization process explains the increase in cross-link density with the increase in irradiation dose [27,29].

The ratio between the cross-linking and scission reactions was evaluated by using the Charlesby–Pinner Equation (5) [30,31].

(5) $$S+\sqrt{S}=\frac{p_{0}}{q_{0}}+\frac{1}{\alpha P_{n}D}$$

where *S* is the sol fraction (*S* = 1 − gel fraction); *p*_0_ is the degradation density (the average number of main chain scissions per monomer unit and per unit dose); *q*_0_ is the cross-linking density (the proportion of monomer units cross-linked per unit dose); *P_n_* is the average degree of polymerization; and *D* is the radiation dose in kGy.

**Figure 2:**
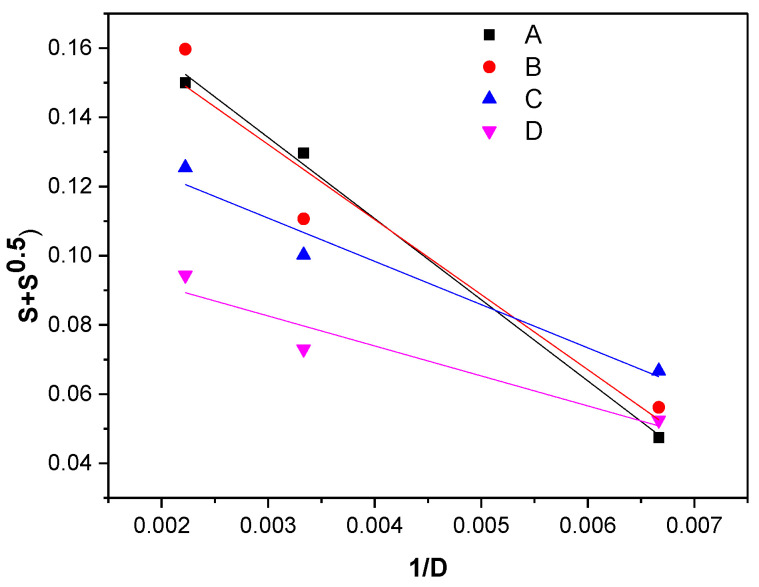
Charlesby–Pinner plots of the composites.

An increase in the *p*_0_/*q*_0_ ratio is associated with the prevalence of scission reactions compared to cross-linking reactions. *p*_0_ represents the degradation degree (average number of chain scissions per monomer unit), and *q*_0_ the cross-link density. The plots of *S* + *S*^1/2^ vs. 1/absorbed dose (1/*D*), from which the ratio *p*_0_/*q*_0_ was calculated, are presented in Figure 2, and the results of the calculations are shown in Table 4.

As can be seen from Table 4, the *p*_0_/*q*_0_ ratio is dependent on the vulcanization accelerator mixture that has been used. An increase in this ratio is associated with an increase in cleavage reaction (degradation) prevalence compared to cross-linking reactions. The order in which the *p*_0_/*q*_0_ ratio increases in the mixtures is D < C < B < A. This result shows that natural rubber vulcanized with sulfur and irradiated with EB degrades less if it contains MBT and CBS, and it degrades better in the presence of MBT and TMTD. Thus, the degradation of the composites in which two primary vulcanization accelerators are used (sulfenamide and thiazole) requires higher irradiation doses than the composites in which one primary and one secondary accelerator (thiourane and thiazole) are used [19].

### 3.2. Mechanical Characteristics

**Figure 3:**
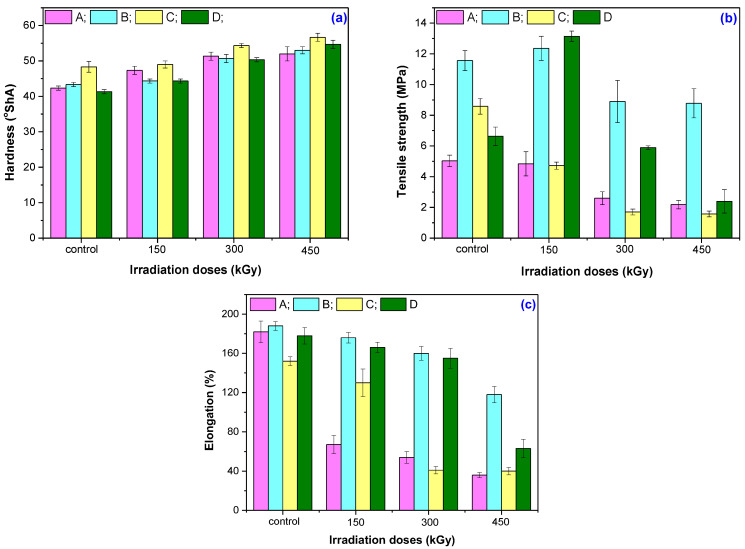
Hardness (**a**), tensile strength (**b**), and elongation (**c**) variation as a function of the electron beam’s irradiation dose.

The influences of accelerator type and irradiation dose on hardness, tensile strength, and elongation of the non-irradiated and irradiated composites were evaluated, and the results are presented in Figure 3a–c.

Table 5 shows the changes (in percentages) in the mechanical properties of the irradiated composites compared to the non-irradiated samples.

For the non-irradiated mixtures, the order in which the vulcanization accelerator mixtures influences the mechanical properties is as follows: D < A < B < C for hardness; A < D < C < B for tensile strength; and C < D < A < B for elongation at break. An increase in the irradiation dose from 150 kGy to 300 and 450 kGy, respectively, leads to a change in the response of the samples in terms of hardness from C < B < D < A to C < B < A < D. Hardness, as a measure of the stiffening of the mixtures, strongly depends on the cross-linking degree: an increase in the cross-linking degree leads to an increase in hardness [32]. Mixture D (MBT + CBS) shows the greatest increase in the cross-linking degree and, consequently, the greatest increase in hardness at 450 kGy.

Irradiation with 150 kGy leads to the improvement in tensile strength from 11.66 MPa to 12.36 MPa for mixture B and from 6.63 MPa to 13.14 MPa for mixture D. An increase in the irradiation dose leads to a deterioration in tensile strength. The order in which the decrease in this mechanical property occurs in the tested mixtures is B < A < D < C, with the greatest decrease being recorded for the mixtures D (MBT + CBS) and C (MBT + TMTD + DPG).

EB irradiation also leads to a decrease in the elongation at break, with mixture A (MBT + TMTD) registering a decrease of 62.97% even at 150 kGy and reaching 80.44% at 450 kGy. The other mixtures record smaller decreases in the elongation at break at 150 kGy (6.38%, 6.74%m, and 14.47% for mixtures B, D, and C, respectively); this changes significantly at the dose of 450 kGy (73.95% and 64.89% for mixtures C and D, respectively). The decrease in the elongation at break at 450 kGy occurs in the order B < D < C < A.

The decreases in the tensile strength and elongation at break are due to an increase in the cross-linking degree. When the cross-link density due to the vulcanization reactions between the vulcanization agents and rubber matrix increases, the average weight of the molecules between the cross-linking points decreases, which explains the decrease in mechanical properties [32,33,34]. Thus, the change in the mechanical properties of the mixtures subjected to irradiation is a consequence of their degradation.

Depending on the amount of sulfur and accelerator in the rubber mixtures, vulcanization systems are classified into three types: conventional (CV), semi-efficient (semi-EV), and efficient (EV). The type of vulcanization system determines the type of bonds that are formed during the vulcanization process, as well as the mechanical properties and the way that the rubber-based materials behave when they are subjected to a degradation process. Thus, during the vulcanization process, poly- and disulphidic cross-links and monosulphidic cross-links are formed in different percentages, depending on the applied vulcanization system (CV, semi-EV, or EV) [35,36,37], as shown in Table 6.

The recipes in Table 2 show that the vulcanization system used to make the mixtures A, B, C, and D is a conventional one (CV), which has a large amount of sulfur compared to the accelerator and offers the possibility of forming more polysulfide bonds. As the concentration of the accelerator increases (from 1 phr in mixtures A, B, and D to 1.5 phr in mixture C), other types of reactions, such as desulphuration or decomposition, may occur. Desulphurization results in the formation of mono- and disulfide bonds, while decomposition leads to the formation of cyclic sulfides and conjugated dienes [35,36,37]. An increase in temperature during irradiation causes the polysulfide bonds to break into mono- and disulfide bonds and leads to a decrease in tensile strength associated with the degradation of the vulcanized material. The energy applied in the irradiation process has the potential to completely or partially break the three-dimensional network, thereby breaking the bonds formed during the vulcanization process, which can be the C-S, S-S, or C-C type [38,39]. It is difficult to highlight a certain selectivity of breaking these bonds or the proportion in which this occurs due to the irradiation with EB of high energy because, although the energy of the beam is sufficient to break the bonds, the binding energies of S-S, C-S, and C-C bonds are very close (227, 273, and 348 kJ/mol, respectively) [38,40]. The change in the mechanical properties of the irradiated mixtures A, B, and C shows that the breaking of the bonds in the three-dimensional network formed in the vulcanization process (S-S, C-S, and C-C) happens randomly without being able to highlight a specific selectivity.

### 3.3. Solvent Uptake Tests and Weight Loss

In the degradation process of elastomeric materials, the bonds formed during their vulcanization process are broken, which leads to the formation of low-molecular-weight compounds that can be removed from the structure by immersion in solvents. The mass loss in the solvent is strictly related to the gel fraction and the cross-linking degree, which we have shown to undergo changes during the degradation process. The fraction of material soluble in the solvent is a measure of degradation degree, i.e., the number of cleavages and low-molecular-weight compounds that are formed and pass into the solvent [41].

**Figure 4:**
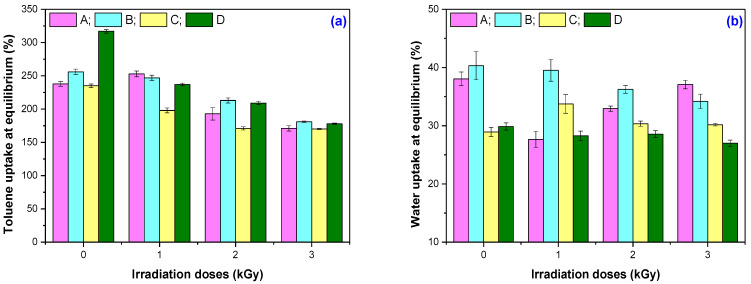
Toluene (**a**) and water (**b**) uptakes at equilibrium.

Figure 4 shows the results regarding the absorption of toluene (Figure 4a) and water (Figure 4b) at a room temperature of 23 ± 2 °C until the equilibrium has been reached by the four mixtures under investigation (6 days in toluene and 100 days in water, respectively). After the equilibrium was reached, the elastomeric samples were air-dried for 6 days and then in a laboratory oven at 80 °C for 72 h, and based on the results, their mass loss was determined. Table 7 presents the mass loss variation of the mixtures after their immersion in both solvents.

As can be seen from Figure 4, the order in which solvent uptake at equilibrium increases for the non-irradiated mixtures is as follows: C < A < B < D in the case of immersion in toluene (a) and C < D < A < B in the case immersion in water (b). Solvent absorption is strongly dependent on the structure and capacity of the polymer matrix and the filler (plasticized starch in this case) to provide ways for the solvent to penetrate the vulcanized material. The presence of voids and the lack of structural uniformity increase the absorption. The more or the larger the voids left in the material after the vulcanization process, the greater the amount of solvent absorbed. The adhesion between the filler and the polymer matrix also contributes to the increase in solvent absorption. The greater the adhesion and, thus, the formation of grafting bonds during the vulcanization process between the plasticized starch and natural rubber, the less favorable the absorption of solvents [42]. Toluene absorption is mostly achieved by the polymer matrix, i.e., natural rubber, and is dependent on its cross-linking degree. As expected, the highest absorption of toluene is achieved by mixture D (MBT + CBS), which has the lowest value of cross-linking degree (see Figure 1b). Water absorption mostly depends on the presence of water-soluble and hygroscopic components in the vulcanized material, as well as on the adhesion between the matrix and the filler [43]. Mixture B (MBT + DPG) shows the highest water absorption.

The absorption of water and toluene in the irradiated samples occurs as follows: at 150 kGy, C < D < B < A (toluene) and A < D < C < B (water); at 300 kGy, C < A < D < B (toluene) and D < C < A < B (water); and at 450 kGy, C < A < D < B (toluene) and D < C < B < A (water). Ionizing radiation forms reactive species (radicals and excited molecules) in the irradiated materials, which modify their molecular structure. These species participate in the splitting reactions of vulcanized molecular chains and are responsible for the appearance of compounds with low molecular weight that can easily pass into solvents. The presence of air during the irradiation of vulcanized elastomeric mixtures leads to the formation of oxidative degradation compounds, such as alcohols, aldehydes, epoxides, ketones, esters, and carboxylic acids [15]. From Figure 4, it can be seen that with an increase in the irradiation dose, the absorptions at equilibrium decrease. In order to not only connect this decrease in the penetration of the solvents into the irradiated materials with the increase in cross-linking degree, the mass loss in both solvents after reaching equilibrium was also calculated (Table 7). Table 7 shows that the mass loss in toluene, when compared to the non-irradiated samples (for each mixture), decreases at the irradiation dose of 150 kGy. This is due to the increase in the cross-linking degree that can be associated with the continuation of the vulcanization process. By further increasing the irradiation dose, the mass loss in toluene increases for all mixtures, reaching higher values than those of the non-irradiated samples at the irradiation dose of 450 kGy. The mass loss in toluene compared to the value obtained for the non-irradiated sample, at the highest dose of irradiation, is 47.05%, 56.25%, 70.43%, and 89.55% for the mixture D, A, B, and C, respectively. The greater the mass loss, the more cleavages of the grafting and cross-linking bonds can be assumed to take place, and all small molecular compounds that are formed are removed in toluene. The fraction of the soluble material is a measure of the degree of degradation, that is, the number of cleavages [41]. With starch being a polysaccharide that is insoluble in water and organic solvents, we can consider that the mass loss in toluene is due to the degradation of the composites due to the electron beam irradiation. The mass loss behavior of the mixtures irradiated at 150 kGy is different in water compared to toluene. Only mixture A records a decrease in mass loss. With an increase in the irradiation dose, all mixtures show mass loss. Mixture A (MBT + TMTD) at 450 kGy has a mass loss almost equal to that of the non-irradiated sample (3.46% for the non-irradiated sample and 3.42% for the irradiated sample). Given its behavior, we can assume that, during vulcanization, there is a more pronounced grafting of the plasticized starch onto the natural rubber chain, a process continued by irradiation, with the mass loss being close to 0% for the dose of 450 kGy. All other mixtures show mass loss after immersion in water for 100 days. At the highest irradiation dose of 450 kGy, the mass losses in water compared to the values obtained for the non-irradiated samples are 33.33%, 44.82%, and 57.55% for mixtures C, D, and B, respectively. The greater the mass loss in water, the weaker the starch grafting and, therefore, the weaker the polymer matrix–filler interaction. The polymer matrix (natural rubber) contains a small percentage of proteins, fatty acids, resins, and natural polymer chains formed by hundreds of individual amino acid residues connected to each other by peptide bonds, which can be removed by immersion in water [44]. Instead, the starch used as a filler is mainly composed of α-D-glucose, amylopectin—a branched component—and amylose—a linear component. Through the action of EB on the starch, are generated free radicals that are capable to produce the decomposition of macromolecules and to create molecules with small chains, i.e., to break the bonds of amylopectin and amylose [45,46].

### 3.4. Structural Investigations by Fourier-Transform Infrared Spectroscopy (FTIR) Technique

**Figure 5:**
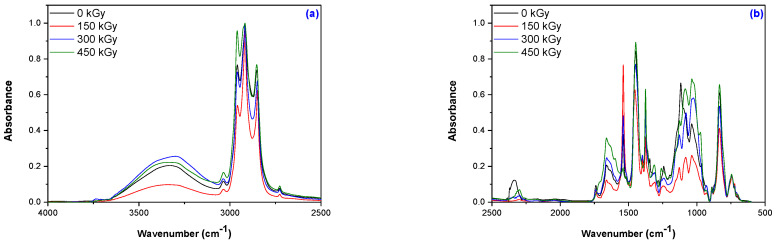

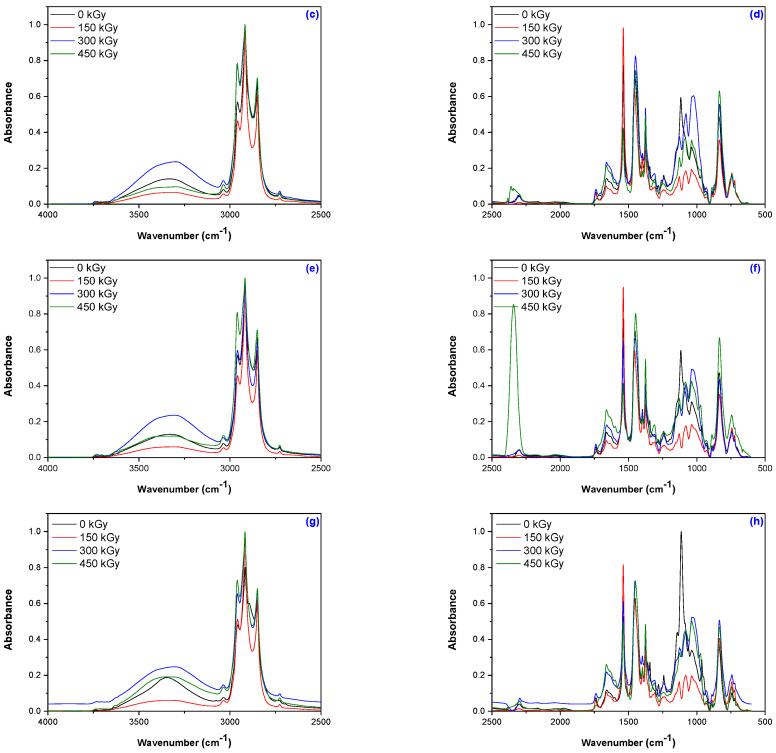
ATR-FTIR spectra of the non-irradiated and irradiated elastomeric composites: mixture A (**a**,**b**), mixture B (**c**,**d**), mixture C (**e**,**f**), and mixture D (**g**,**h**) in the range of 4000–650 cm^−1^.

The results obtained from the ATR-FTIR analysis, which was performed on the non-irradiated and irradiated samples in order to highlight the degradation of the latter through the characteristic absorptions of cleavage and oxidation reactions, are presented in Figure 5a–h. The splitting of the vulcanization bonds is highlighted by the changes in the absorption bands that are characteristic of the vulcanization process (C-C, C-S-C, -C-S_2_-C-, or C-S_x_-C bonds). The same splitting reactions should lead to the appearance of methyl (-CH_3_)- or tertbutyl (-C-(CH_3_)_3_)-type absorption bands due to the homolytic or heterolytic dissociations. Oxidation reactions are highlighted by the appearance of bands that are characteristic of oxidation products (alcohols, aldehydes, ketones, and carboxylic acids). All these products obtained from splitting or oxidation are degradation products.

The spectra of the tested composites show broad absorptions in the range of 3700–3034 cm^−1^ due to the presence of N-H stretching vibrations, which provides information about hydrogen bonding in proteins, mono- and di-peptides from natural rubber [47,48,49,50]. The absorptions between 3350 and 3325 cm^−1^ are due to the -OH functional group from the starch, and the band amplitude indicates the presence of inter-molecular hydrogen bonds [51]. With an increase in the irradiation dose, due to the continuation of the vulcanization process, there is an increase in absorption only for mixture A, while in all other mixtures, it decreases due to the oxidation process [52]. The intensity of the absorption bands due to the formation of the hydroxyl group (-OH) as a result of degradation through oxidation reactions increases in the range of 3326–3035 cm^−1^ at the irradiation doses of 300 and 450 kGy [22,53,54,55]. At the same time, these absorptions can also be due to the stretching vibration of the hydroxyl group (OH) linked to the hydrogen in the polysaccharides, with the increase in absorption in this area also being due to the degradation of starch [56]. The absorptions in the range of 3034–3036 cm^−1^ correspond to the stretching of CH from the -CH=CH_2_ group. In this interval, irradiation at a dose of 150 kGy leads to a decrease in the absorption of all mixtures as a result of the continuation of the vulcanization process. This situation is changed with increasing radiation dose, when cleavage reactions and groups specific to the degradation process begin to appear [57]. The highest absorption in this interval is that of mixture D (MBT + CBS), followed by C, B, and then A. The bands that are characteristic of sp3 saturated aliphatic C–H bonds appear between 2960 and 2842 cm^−1^ and are attributed to -CH_3_ asymmetric stretching vibration (2960–2957 cm^−1^), -CH_2_- asymmetric stretching vibration (2919–2917 cm^−1^), and -CH_2_- symmetric stretching vibration (2853–2847 cm^−1^), respectively [58]. These are due to both polymer matrix and vulcanization accelerators. EB irradiation leads to notable changes at 2960–2957 cm^−1^ and 2853–2847 cm^−1^ and less important changes in the region 2919–2917 cm^−1^. The association of an increase in absorption bands with composite degradation by EB irradiation is supported by the results obtained from the physical and mechanical tests. The biggest increases in absorption in these regions were recorded for mixture D, followed by C, B, and A.

In the region of 2730–2725 cm^−1^ are the specific absorption bands of the alkyl C–H stretches from aldehydes (R-CHO) [59]. These absorption bands are highlighted in the non-irradiated samples due to the oxidation products formed during the vulcanization of mixtures A, B, and C, but they also in appear in mixture D, even if after irradiation only. The order in which this absorption increases with an increase in the irradiation dose is B < A < C < D. The absorption bands in the range 1738–1740 cm^−1^ that are highlighted in all non-irradiated samples are due to the presence of fatty acids and their esters. For mixtures A and B, they decrease, and for mixtures C and D, they increase, with the largest increase seen for mixture D. The absorptions that increase with an increase in the irradiation dose to 300 and 450 kGy in the case of mixtures C and D can be attributed to the carbonyl groups (-C=O) from ketone (R_2_C=O) or aldehyde (RCOH), being a result of the oxidative degradation process that occurs during irradiation in the presence of oxygen [53,60,61,62].

In the region of 1653–1649 cm^−1^, absorptions are present in all non-irradiated samples, and these absorptions increase with an increase in the irradiation dose. They can be due to stretching vibrations -C=C- in the structure of the natural rubber matrix but also to the formation of oxidative degradation products, such as carboxylate groups or conjugated ketone (>C=O) [22,61]. Additionally, in this area, absorptions can be due to primary (NH-R) or secondary (NH < R_2_) amines from the decomposition of the vulcanization accelerators [63].

The absorptions recorded in the range of 1540–1538 cm^−1^ may correspond to aromatic rings from the vulcanization accelerators and can be correlated with the presence of weak absorptions in the region 3150–3000 cm^−1^ corresponding to C-H stretch. Although the absorption changes in this area are reduced even with an increase in the irradiation dose, they can be associated with possible splitting reactions of the aromatic rings in the vulcanization accelerators. The order of the increase in absorptions in this interval is A < D < C < B [63]. The bands observed here can also be attributed to the C-S bond formed during the vulcanization process, which seems to be broken by irradiation [64].

The absorptions in the regions of 1445–1398 cm^−1^ and 13,776–1375 cm^−1^ can be due to CH_2_ deformation, an asymmetric stretching of CH_3_ that is characteristic of natural rubber [22,65], but also to the amide groups (-NH_2_) in the vulcanization accelerators. With an increase in the irradiation dose, the absorptions in these regions increase, which can lead to the conclusion that the vulcanization accelerators in the presence of sulfur continue the vulcanization process or form compounds with small molecules (this may be argued by the decrease in the mechanical properties and the increase in the cross-linking degree) [66].

The absorption bands located between 1320 and 1280 cm^−1^ highlighted in the non-irradiated samples are due to CH_2_ and CH_3_ deformation vibrations in natural rubber [67]. By irradiation, these groups undergo oxidation reactions with the formation of carboxylic compounds, aldehydes, or ketones or the breaking of molecular chain through the cleavage of internal bonds [61,68,69]. Carboxylic compounds show a strong band in the region 3300–2500 cm^−1^ due to the formation of the –OH group and the stretching of the C=O and C-O groups in the regions 1760–1690 cm^−1^ and 1320–1210 cm^−1^. In the region of 1320–1280 cm^−1^, the absorption bands increase with an increase in the irradiation dose due to the formation of some degradation compounds by irradiation, including compounds containing the C–O group [65]. The order of the increase in these bands for the vulcanization accelerator mixtures is D < B < A < C.

The absorption bands present in the non-irradiated samples in the range of 1260–1240 cm^−1^ can be attributed to the impurities present in natural rubber (proteins, lipids, amino acids, and peptides) that are unstable and can be easily degraded by irradiation [65,70]. With an increase in the irradiation dose, the absorptions of these bands are reduced. The stretching vibration of C-N groups in aliphatic amines is found in the range of 1250–1020 cm^−1^. Their absorptions decrease in intensity for mixtures B, C, and D (the order being C < B < D) with an increase in the irradiation dose due to the decomposition of the vulcanization accelerators [59]. The strong absorption bands located between 1085 and 1080 cm^−1^ that are not found in the non-irradiated samples may be due to the vibrations of the S-O bonds formed as a result of the oxidative degradation reactions [13]. The oxidative degradation processes that take place at the irradiation dose of 450 kGy lead to the increase in these absorption bands in the order B < D < C < A. The formation of the sulfoxide S=O group during the vulcanization process leads to the appearance of some bands in the range of 1035–1027 cm^−1^ in the non-irradiated samples [71]. In their case as well, with an increase in the irradiated dose, the absorptions are intensified in the order B < C < D < A as a result of the oxidative degradation. In the regions 920–975 cm^−1^ and 895–870 cm^−1^, absorptions are recorded for the non-irradiated samples due to the presence of vinyl terminal groups (-CH=CH2) and double olefinic bond that binds the simple vinyl groups (C=CH_2_) formed during vulcanization [13,63]. With an increase in the irradiation dose, these absorptions also increase due to the splitting/degradation reactions. The absorptions in the regions of 835–832 cm^−1^ and 747–743 cm^−1^ can be due to para- and ortho-aromatic compounds from the vulcanization accelerators [63]. The modification of these bands due to irradiation, depending on the type of accelerator used for vulcanization, occurs in the order A < D < B < C in the range of 835–832 cm^−1^ and B < A < D < C in the range of 747–743 cm^−1^. The structural changes in the irradiated samples, as highlighted by the FTIR analysis, are associated with the appearance of products specific to the cleavage or oxidation reactions (carboxyl compounds, aldehydes, or ketones). These, along with the changes in the physico-chemical properties (gel fraction, degree of reticulation, mechanical properties, and mass loss in solvents) support the idea of inducing the degradation of elastomeric products by electron beam irradiation at doses over 150 kGy.

### 3.5. Morphological Investigations by Scanning Electron Microscopy (SEM) Technique

**Figure 6:**
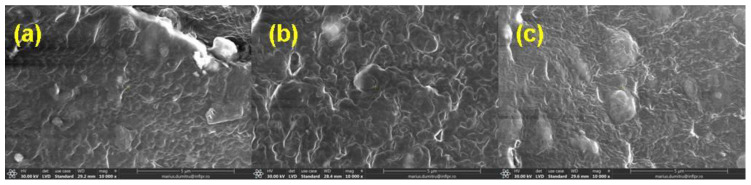
SEM micrographs of mixture A at magnification of 10,000: (**a**) non-irradiated and irradiated at (**b**) 150 kGy and (**c**) 450 kGy.

**Figure 7:**
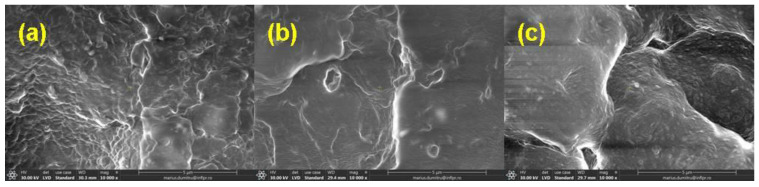
SEM micrographs of mixture B at magnification of 10,000: (**a**) non-irradiated and irradiated at (**b**) 150 kGy and (**c**) 450 kGy.

**Figure 8:**
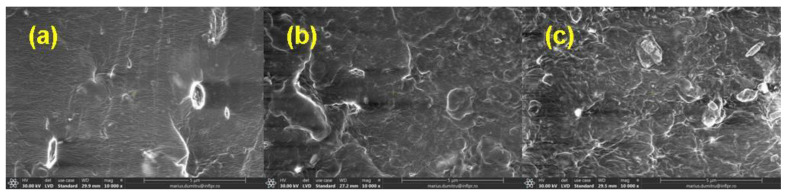
SEM micrographs of mixture C at magnification of 10,000: (**a**) non-irradiated and irradiated at (**b**) 150 kGy and (**c**) 450 kGy.

**Figure 9:**
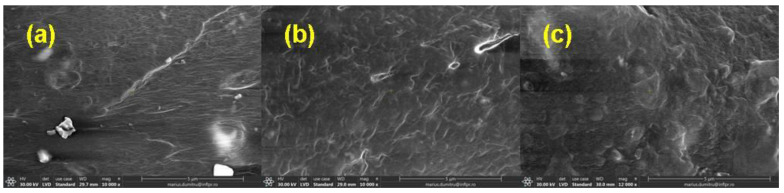
SEM micrographs of mixture D at magnification of 10,000: (**a**) non-irradiated and irradiated at (**b**) 150 kGy and (**c**) 450 kGy.

The morphological changes in mixtures A, B, C, and D before and after irradiation with 150 kGy and 450 kGy are shown in Figure 6, Figure 7, Figure 8 and Figure 9.

The SEM images were acquired on the samples dried in air for six days and then in a laboratory oven at 80 °C for 72 h in order to avoid the interference with possible particles of the unreacted material (in the non-irradiated samples) or with fragments of low molecular weight that result from the degradation process induced by exposure to ionizing radiation (in the irradiated samples).

In Figure 6a, Figure 7a, Figure 8a and Figure 9a, the presence of the filler and its agglomerations can be observed, which may indicate a weak interaction between the polymer matrix and the filler as a result of the vulcanization process. Filler particles (plasticized starch) and imperfections (waves) due to the vulcanization process are observed on all investigated surfaces [72].

In Figure 6b,c, Figure 7b,c, Figure 8b,c and Figure 9b,c, a change in the surfaces of the irradiated samples can be observed, with the matrix–filler connection appearing to be impaired (cracks and micro-voids) [72,73]. This result suggests that the interface adhesion in these composites decreases even more with an increase in the irradiation dose. Therefore, the investigated sulfur vulcanized materials (regardless of the type of vulcanization accelerator used) are degraded by irradiation, a result that is also supported by the other presented results, namely the modifications in the physico-chemical, mechanical, and structural properties.

## 4. Conclusions

Elastomeric composites based on natural rubber and plasticized starch were obtained by sulfur vulcanization, with the cross-linking processes being driven by four mixtures of vulcanization accelerators (MBT + TMTD, MBT + DPG, MBT + DPG + TMTD, and MBT+ CBS). The degradation experiments of the composites were carried out by irradiation with a 5.5 MeV electron beam at the irradiation doses of 150, 300, and 450 kGy in order to establish a connection between the mixture type, irradiation dose, and modification in composite properties.

The decrease in the gel fraction with increasing irradiation dose was accompanied, as it was expected, by an increase in the cross-link densities. The most sensitive composite, from this point of view, was the one containing a medium-fast primary accelerator (MBT) and a secondary accelerator (CBS) at the irradiation dose of 450 kGy. The degradation process was determined through the ratio between the cross-linking and scission/cleavage reactions (*p*_0_/*q*_0_). The results showed that under irradiation, the composites cross-linked with a combination of a medium-fast primary vulcanization accelerator and a secondary accelerator degraded less than those cross-linked with a combination of a medium-fast primary vulcanization accelerator and an ultrafast secondary accelerator. Thus, composites cross-linked using a primary vulcanization accelerator (sulfenamide or thiazole) requires higher irradiation doses than those cross-linked using a primary and a secondary vulcanization accelerator (thiourane or thiazole). The mechanical properties were evaluated before and after irradiation. As it was expected, the biggest changes were registered at the irradiation dose of 450 kGy by the composite cross-linked with a combination of a primary vulcanization accelerator and a secondary one (MBT + CBS). With an increase in the irradiation dose, all composite mixtures showed mass loss regardless of the vulcanization accelerators’ combination. It looks like the combination of a primary and a secondary accelerator leads to the formation of structures that are easier to degrade by electron beam irradiation. The degradability induced by electron beam irradiation was determined by the ATR-FTIR analysis, which showed specific groups of oxidative reactions associated with degradation in the samples irradiated at high doses. The morphological investigations by SEM showed cracks and micro-voids on the irradiated surfaces as a result of the degradation process.

## Figures and Tables

**Table 1:** Physical and chemical characteristics of the vulcanization accelerators.

Accelerator	Group/Speed	Use	Chemical Structure	Property
MBT (2-Mercaptobenzothiazole)	Thiazoles/Scorchy. Ultra-fast	Primary accelerator	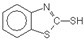	molecular weight: 167.2 melting point: 177–182 °C boiling point: 302 °C density: 1.42 g/cm^3^
CBS (N-Cyclohexyl-2-benzothiazole sulfonamide)	Sulfenamides/Delayed action. Ultra-fast	Primary accelerator	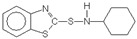	molecular weight: 264.4 melting point: 97–105 °C density: 1.31 g/cm^3^
DPG (Diphenyl guanidine)	Guanidines/Scorchy and slow cure rate	Primary and secondary accelerators	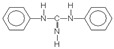	molecular weight: 211.27 melting point: 148 °C density: 1.15 g/cm^3^
TMTD (Tetramethylthiuram disulfide)	Thiurams/Ultra-fast	Primary/secondary accelerator, and sulfur donor	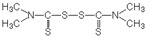	molecular weight: 240.4 melting point: 155–158 °C boiling point: 129 °C density: 1.43 g/cm^3^

**Table 2:** The recipes used in the obtained composites.

Ingredients (phr)	Mixture Codes			
	A	B	C	D
NR	95	95	95	95
Maleated NR (NR-g-MA)	5	5	5	5
Plasticized starch	30.8	30.8	30.8	30.8
ZnO	5	5	5	5
Stearic acid	0.5	0.5	0.5	0.5
PEG 4000	3	3	3	3
Sulfur	2.5	2.5	2.5	2.5
Antioxidant 4010 (g)	1	1	1	1
DPG	-	0.5	0.5	-
MBT	0.5	0.5	0.5	0.5
TMTD	0.5	-	0.5	-
CBS	-	-	-	0.5

**Table 3:** Modifications of gel fraction and cross-link density percentages of the irradiated mixtures, reported to be corresponding to the non-irradiated sample.

Doses (kGy)	Gel Fraction (%)				Cross-Link Density (%)			
	A	B	C	D	A	B	C	D
150	+0.92	+0.87	+0.28	+0.26	+5.85	+9.50	+37.09	+68.55
300	−0.23	+0.14	−0.17	+0.05	+44.66	+46.80	+74.66	+110.28
450	−0.64	−0.82	−0.60	−0.24	+77.20	+83.33	+74.45	+179.76

**Table 4:** *p*_0_/*q*_0_ ratio of the composites.

Mixture Type	*p_0_*/*q_0_* Ratio
A (MBT + TMTD)	0.2045
B (MBT + DPG)	0.1972
C (MBT + DPG + TMTD)	0.1484
D (MBT + CBS)	0.1036

**Table 5:** Percentage modifications of mechanical properties of the samples after irradiation aging.

Irradiation Dose (kGy)	Mechanical Properties			
	A	B	C	D
	*Hardness (%)*			
150	+11.81	+2.31	+1.38	+7.26
300	+21.26	+16.92	+12.41	+21.77
450	+22.83	+22.31	+17.24	+32.26
	*Tensile strength (%)*			
150	−3.74	+6.06	−44.96	+98.26
300	−48.36	−23.65	−80.13	−11.07
450	−56.67	−24.66	−81.75	−63.99
	*Elongation (%)*			
150	−62.97	−6.38	−14.47	−6.74
300	−70.44	−14.89	−73.29	−12.92
450	−80.44	−37.23	−73.95	−64.89

**Table 6:** Components used in conventional (CV), semi-efficient (semi-EV), and efficient (EV) vulcanization systems and vulcanizates’ structures (cross-link type).

Components (phr) and Vulcanizate Structures (%)	Vulcanization Systems		
	CV	Semi-EV	EV
**Component used**			
Sulfur (phr)	2.0–3.5	1.0–1.7	0.4–0.8
Accelerators (phr)	1.2–0.4	2.5–1.2	5.0–2.0
Accelerators/sulfur ratio	0.1–0.6	0.7–2.5	2.5–12.5
**Cross-link type**			
Poly- and disulphidic cross-links (%) -C-S_x_-C- and -C-S_2_-C-	95	50	20
Monosulphidic cross-links (%) -C-S-C-	5	50	80
Cyclic sulphide concentration 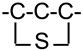	high	medium	low

**Table 7:** Mass loss of the mixtures in toluene and water.

Irradiation Dose (kGy)	Mixtures			
	A	B	C	D
	Mass loss in toluene (%)			
0	1.12 ± 0.01	1.15 ± 0.06	0.67 ± 0.02	0.51 ± 0.01
150	0.21 ± 0.06	0.28 ± 0.14	0.39 ± 0.28	0.25 ± 0.08
300	1.35 ± 0.60	1.01 ± 0.45	0.84 ± 0.67	0.47 ± 0.33
450	1.75 ± 0.09	1.96 ± 0.05	1.27 ± 0.04	0.75 ± 0.19
	Mass loss in water (%)			
0	3.43 ± 0.06	2.78 ± 0.33	1.47 ± 0.09	1.45 ± 0.01
150	2.65 ± 0.21	3.65 ± 0.19	1.52 ± 0.30	1.76 ± 0.18
300	3.15 ± 0.24	3.79 ± 0.24	2.06 ± 0.12	1.81 ± 0.07
450	3.42 ± 0.13	4.38 ± 0.48	1.96 ± 0.02	2.10 ± 0.16

## Data Availability

Data sharing is not applicable.
